# Metabolic brain imaging with glucosamine CEST MRI: in vivo characterization and first insights

**DOI:** 10.1038/s41598-023-48515-5

**Published:** 2023-12-12

**Authors:** Michal Rivlin, Or Perlman, Gil Navon

**Affiliations:** 1 School of Chemistry, Tel-Aviv University, Tel-Aviv, Israel; 2 Department of Biomedical Engineering, Tel-Aviv University, Tel-Aviv, Israel; 3 Sagol School of Neuroscience, Tel-Aviv University, Tel-Aviv, Israel

**Keywords:** Biomarkers, Chemistry

## Abstract

The utility of chemical exchange saturation transfer (CEST) MRI for monitoring the uptake of glucosamine (GlcN), a safe dietary supplement, has been previously demonstrated in detecting breast cancer in both murine and human subjects. Here, we studied and characterized the detectability of GlcN uptake and metabolism in the brain. Following intravenous GlcN administration in mice, CEST brain signals calculated by magnetization transfer ratio asymmetry (MTRasym) analysis, were significantly elevated, mainly in the cortex, hippocampus, and thalamus. The in vivo contrast remained stable during 40 min of examination, which can be attributed to GlcN uptake and its metabolic products accumulation as confirmed using ^13^C NMR spectroscopic studies of brain extracts. A Lorentzian multi-pool fitting analysis revealed an increase in the hydroxyl, amide, and relayed nuclear Overhauser effect (rNOE) signal components after GlcN treatment. With its ability to cross the blood-brain barrier (BBB), the GlcN CEST technique has the potential to serve as a metabolic biomarker for the diagnosis and monitoring various brain disorders.

## Introduction

Metabolic changes are increasingly recognized as key players in brain diseases and may help reveal new approaches for diagnosis and treatment. Currently, the early detection of preclinical brain disorders remains a challenge. Diagnostic processes could greatly benefit from imaging methods capable of identifying abnormal changes in brain metabolism. These strategies hold potential for assessing therapeutic effectiveness during both pre-clinical and clinical stages of diseases. Consistent with its critical role for physiological brain function, disruption of normal glucose (D-Glc) metabolism forms the pathophysiological basis for many brain disorders^1^. Accordingly, positron emission tomography (PET) technology has enabled the examination of brain metabolism by detecting the uptake of a radioactive D-Glc analog 2-fluoro-2-deoxy-D-glucose ([18F] FDG). This technology has proven valuable for detecting brain tumors and their metastases^2^, as well as differentiating between AD and other neurodegenerative diseases^3^. However, when it comes to wide screening tests for population risk assessment at the early stages of pathology or extensive follow-up investigations, PET imaging has several limitations. It has low spatial resolution and requires expensive special infrastructures to synthesis, distribute, and dispose of radioactive tracers. Furthermore, ionizing radiation limits repeated measurements.

Chemical exchange saturation transfer (CEST) MRI is an increasingly investigated imaging technique that enables the non-invasive measurement of metabolic activity in living organisms. Monitoring the uptake and metabolism of D-Glc in the brain may be useful for clinical assay. The CEST MRI technique has been used to measure D-Glc uptake in the animal and human brains to study various pathological conditions including tumors^4–6^, traumatic brain injury^7^, Alzheimer's disease^8^, and hypoxia^9^. However, the D-Glc CEST signal is relatively low and drops rapidly after the agent administration, which is quickly converted to lactic acid by cells in both brain^10^ and tumor models^11^, limiting its imaging efficiency. Thus, CEST imaging of the D-Glc *analogs* uptake has been employed to replicate cancer research using animal models^12,13^. These include 2-deoxy-D-glucose (2DG)^11,14^, FDG^14^, 3-O-methyl-D-glucose (3OMG)^15–17^, and N-acetyl-D-glucosamine (GlcNAc)^18,19^. It was recently demonstrated that the CEST-MRI approach may be utilized to detect 2DG absorption in different regions of the brain of an AD mouse (APP23 model)^20^. The CEST method was used to show that 20-month-old APP23 mice could be differentiated from healthy WT mice by reduction in 2DG brain absorption. Differences in brain D-Glc metabolic activity can be noticed within minutes of injecting 2DG into the cortical area. Chemical exchange spin lock (CESL) and CEST are both capable of measuring identical chemical exchange-related signals of introduced D-Glc or its analogs. The 3OMG-CESL approach was recently demonstrated in an animal model to categorize distinct ischemic regions in acute stroke^21^. However, doses of 3 g/kg of 3OMG in animals cause depression of the central nervous system, which may limit the level of the 3OMG CEST signal for clinical usage (Horizon 2020 GLINT consortium report: https://cordis.europa.eu/project/id/667510/reporting). However, because the toxicity of 2DG^22^ and 3OMG at high concentrations precludes their usage in laboratory animals, it is necessary to use nontoxic sugars to expedite the conversion of CEST-MRI to clinical brain diseases diagnosis.

Recently, we suggested the use of CEST-MRI of glucosamine (2-amino-2-deoxy-D-glucose, GlcN) for the detection of cancer in both animal and human studies^18,23,24^. Unlike D-Glc which is rapidly metabolized to lactic acid, the GlcN CEST signal in breast cancer tumors persists for long time periods^18^. GlcN is known to cross the blood-brain barrier (BBB)^25^, which renders it as a feasible and attractive agent for studying brain metabolism and uptake. Changes in GlcN accumulation would thus reflect variations in brain metabolic activity. GlcN is bio-compatible and widely used for relieving arthritic complaints. In the US, it is one of the most common non-vitamins, non-mineral, food supplements used by adults. GlcN is usually taken orally and 90% of it is absorbed in humans. Some studies suggest that GlcN has no effect on blood Glc levels or insulin sensitivity, making it suitable for a broader population, including diabetics and individuals who are sensitive to Glc variations^26,27^.

The aims of this study were: (i) To evaluate the ability to quantify GlcN uptake and metabolism in the mouse brain using the CEST MRI technique, (ii) to determine the sensitivity of the GlcN CEST signal as measured by different analysis metrics, such as the common magnetization transfer ratio asymmetry (MTRasym) and the multi pool Lorentzian fitting; and (iii) determining the source of GlcN CEST MRI signal in the brain by conducting ^13^C NMR spectroscopy measurements on brain extracts following the administration of [UL-^13^C]-GlcN.

## Methods

### Chemicals

GlcN·HCl and GlcN sulfate were obtained from Sigma-Aldrich (Israel). [UL-^13^C]- GlcN·HCl was obtained from Omicron Biochemicals (USA).

### Animal preparation

All experiments with animal models were carried out in compliance with the principles of the Israel National Research Council (NRC) and were approved by the Tel Aviv University Institutional Animal Care and Use Committee (IACUC) (TAU-MD-IL-2303-116-2). This study is reported in accordance with the ARRIVE guidelines. For the mouse brain scanning, female ICR mice (3-month-old healthy mice, ~20 g) were purchased from ENVIGO RMS (Israel).

In vivo MRI of small animals often necessitates anesthetic to prevent motion during image acquisition. However, anesthetic drugs vary in their capacity to interfere with homeostatic mechanisms responsible for Glc metabolism, confounding effects on Glc kinetics^28–36^. Jin T et al.^37^ demonstrated higher 2DG CESL response at lower isoflurane levels and attributed it to an increase in Glc metabolism associated with reduced anesthesia. To achieve reliable experimental data, the side effects of anesthetics should be eliminated. Physical restriction of the animals allows them to be scanned while awake, but in this case, the influence of stress on the kinetics is another confounding factor that is difficult to quantify and is known to affect brain activity^38^. Thus, in the present work a pre-treatment of Midazolam (2 mg/kg, SC)^39^, was applied as it causes muscle relaxation^39–41^. This allowed the use of a low and stable level of 0.8% isoflurane.

CEST MRI scans were performed before (baseline) and after intravenous (IV) injection of GlcN sulfate (2.5 g/kg, IV, dissolved in saline) following at least 4 hours of fasting with water access. GlcN solutions were prepared a few hours before the experiment to ensure working at a steady state^42^. The respiration rate was monitored using a small animal physiological monitoring system. Animals’ body temperature was maintained at 37 °C.

### Magnetic resonance imaging

All imaging experiments were conducted using a 7 T MRI scanner with a 30-cm bore size, adapted with a Bruker Paravision software (PV6) (Bruker Biospin, Germany). Mice brains were scanned using a quadrature coil (Bruker Biospin, Germany). The FOV was 19 × 19 mm^2^, a matrix of 64 × 64 pixels, and the slice thickness of 1 mm. Z‐spectra was obtained using a single slice, single-shot CEST spin-echo (SE) EPI readout due to its favorable spatiotemporal resolution and scan time^43–45^, employing a saturation pulse power (B_1_) of 2.5 μT, and a CW saturation pulse duration (T_sat_) of 2s, TE/TR = 20/8000 ms, and saturation frequency offsets of + 7 to − 7 ppm with paired alternately 0.25 ppm increments: total scan time for each Z spectra was 7:44 min. Z spectra were acquired before and at least three time periods after GlcN administration (with average times of 15, 23, and 38 minutes post injection). For the calculation of the static magnetic field, a B_0_ map was acquired.

### MRI data analysis

Data was processed in MATLAB using custom-written scripts. . To minimize potential static field inhomogeneity effects, a B_0_ field map was acquired, and 1st and 2nd order shims adjusted using the MAPSHIM routine. Post-processing of Z-spectra included frequency shift correction using the separately acquired B_0_ maps^46^. All analyses were performed on a voxels-wise basis, and the data is presented as the mean ± standard deviation for a fixed-area region of interest (ROI). The following analysis was used:

1. MTRasym=(S^−Δω^ − S^+Δω^)/S_0_, where S^±Δω^ is the signal measured with saturation at ± the relevant solute chemical shift and S_0_ is the unsaturated signal.
2. A multi-pool Lorentzian fitting of the Z-spectra was applied to estimate the CEST effect as obtained by the different pools^46,47^. The Z-spectrum was fitted as the sum of multiple Lorentzian functions with the following equation:
  1 $$1 - \frac{I}{{I_{0} }} = \sum\limits_{i = 1}^{N} {\frac{{A_{i} }}{{1 + 4\left( {\frac{{w - w_{i} }}{{\sigma_{i} }}} \right)^{2} }}} ,$$
  where ω is the frequency offset from the water resonance, and A_i_, ω_i_ and σ_i_ are the amplitude, frequency offset and linewidth of the CEST peak for the ith proton pool, respectively. The integrals of the different pools were calculated as follows: π/2A_i_σ_i_^23^.

### Preparation of brain extracts

The methanol-chloroform-water extraction procedure^48^ was used for producing extracts from mice brains (n=20): brains were surgically excised, weighed, and immediately immersed in liquid nitrogen. The frozen brains were homogenized using a tissue homogenizer, following the methanol-chloroform-water procedure with a volume ratio of 2/2/1.8, respectively. Only the upper aqueous phase was kept for analysis after centrifugation (− 4 °C, 4000 *g*, 15 min.). The samples were gently evaporator dried, then frozen at 80 °C and lyophilized for 24 hours to achieve dryness. Samples were combined and dissolved in 0.6 ml H_2_O containing 5% D_2_O (99.98%, Biolab, Israel), their pH was adjusted to 7.4 and then inserted in a 5 mm tube for ^13^C NMR analysis.

### NMR spectroscopy of brain extracts

^13^C NMR spectra of the extracts were recorded at 125.76 MHz in 5 mm tubes using a Bruker AVANCE3 11.7 T spectrometer. The following acquisition parameters were used: a relaxation delay of 4 s, an acquisition time of 0.6 s, a spectral width of 26 KHz, a data size of 32 K, and a pulse width of 5.5 s (45° flip angle). The data was recorded and processed using Bruker's TOPSPIN 4.2 software.

### Statistical analysis

The one-way analysis of variance (ANOVA) including the Tukey HSD comparisons test was used to calculate statistical significance as obtained before and after GlcN treatment. P-values < 0.05 were considered significant.

## Results

**Figure 1:**
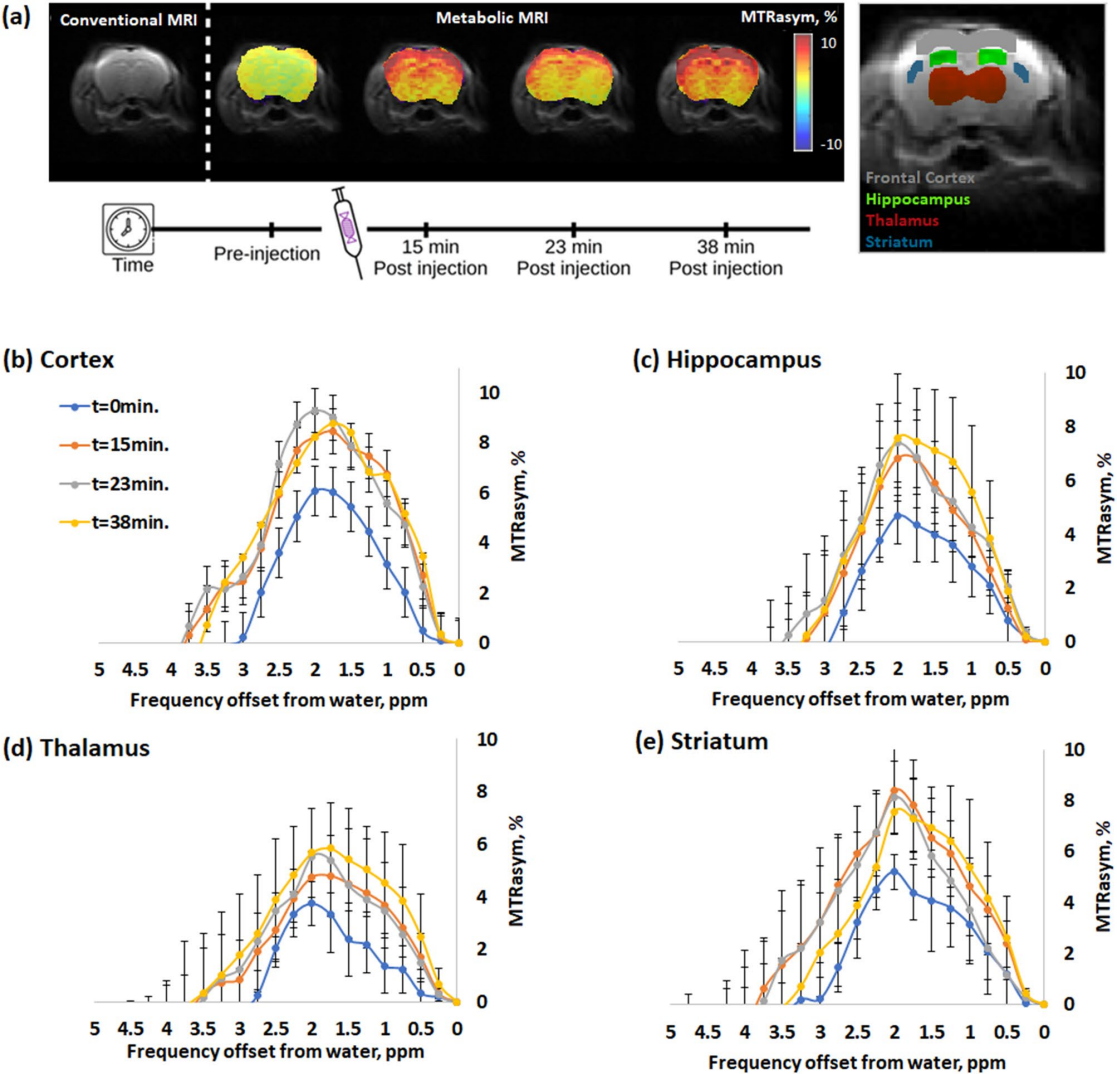
(**a**) T_2_ (SE EPI) image of a representative mouse brain (left), followed by dynamic MTRasym maps (%, at 1.5ppm) before and after GlcN injection (2.5 g/kg, IV) at four time points. The brain with ROIs used is shown on the right. (**b**) MTRasym dynamic time curves of for the brain’s Cortex, (**c**) Hippocampus, (**d**) Thalamus, and (**e**) Striatum (7T, N=6).

The *in vivo* experiments were carried out on female healthy mice brains. All examined mice (N=6) showed significant CEST effects lasting for at least 40 minutes following administration of GlcN. Fig. 1a shows MTRasym maps at 1.5 ppm of dynamic CEST MRI for a representative mouse before and after GlcN treatment (2.5 g/kg, IV). Fig. 1b–e shows the MTRasym signal dynamics in the cortex, hippocampus, thalamus, and striatum, demonstrating an increased MTRasym signal with a maximum around the frequency offset of 1.5-2.2 ppm. During the measurement period (~40 min.), MTRasym signals were sustained in the cortex, hippocampus, thalamus, and striatum of the brain. The in vivo MTRasym profile exhibited an expansion of the overall CEST profile for the different examined parts of the brain (compared to the CEST shape of GlcN solutions^18,23^.

**Figure 2:**
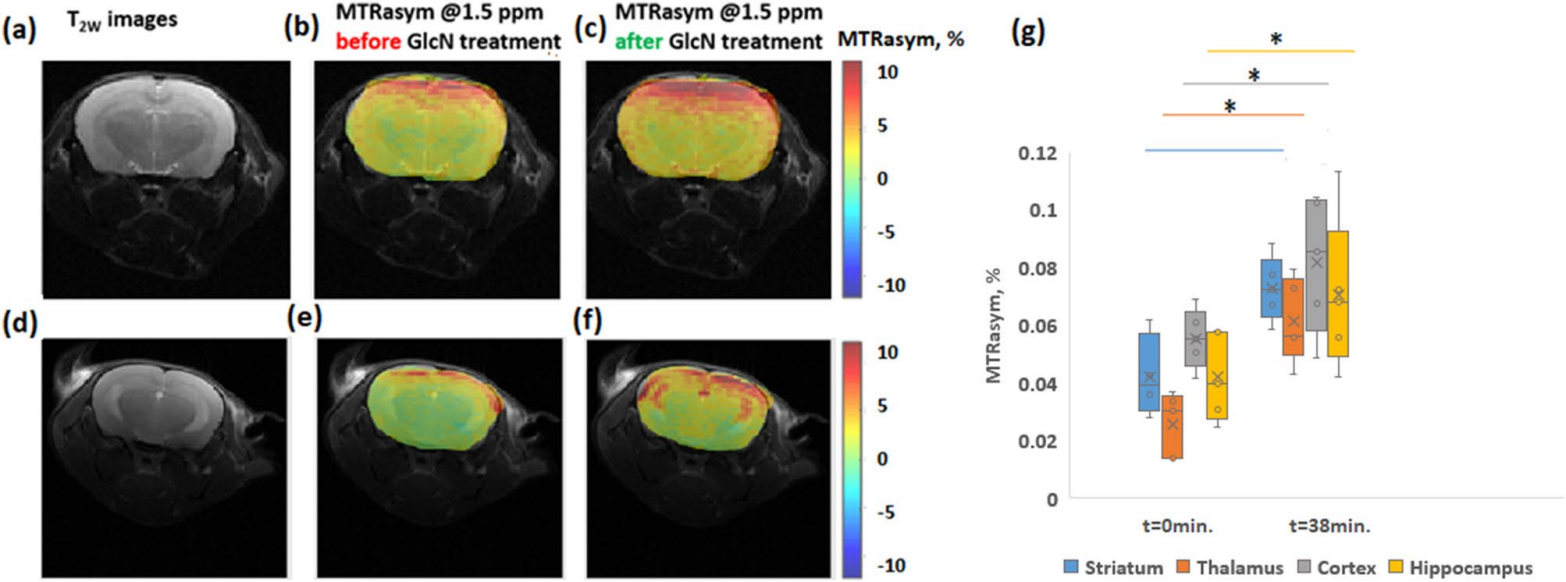
(**a**,**d**) T_2_ anatomical images of two mice brains and their overlay CEST MTRasym maps generated at frequency offset of 1.5 ppm (**b**,**e**) before and (**c**,**f**) after GlcN treatment (2.5 g/kg, IV). (**g**) MTRasym plots (%, at 1.5 ppm) in different brain regions. (The circle in the box plot indicates the MTR value, while x is the averaged value). Significant changes between baseline (control) and 38 minutes after administration are indicated with an asterisk for P<0.05. (N = 6).

**Figure 3:**
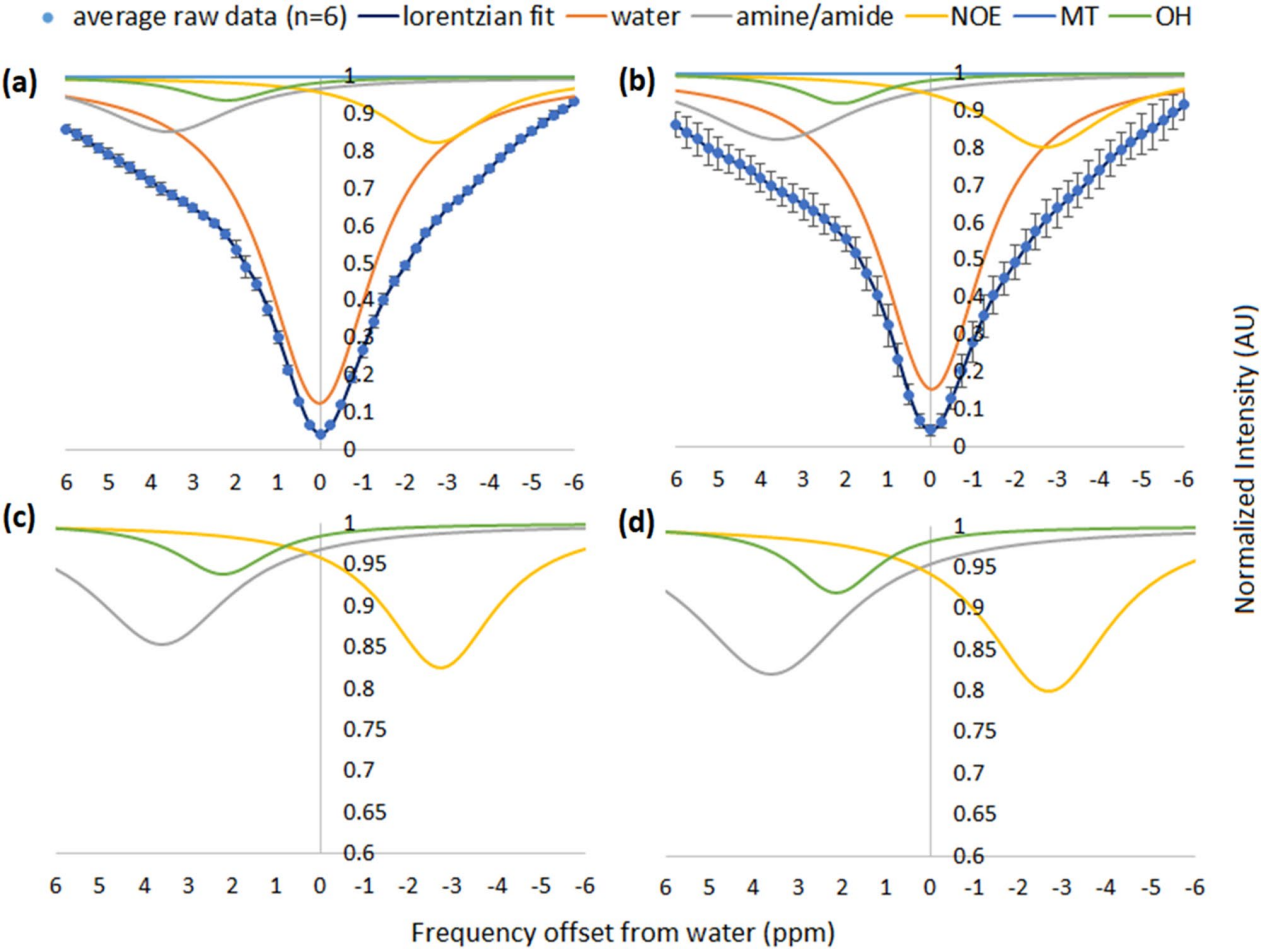
Z-spectra and multi-pool Lorentzian fitted compound contributions obtained from the cortex of mice (n=6) at 7T (**a**) before and (**b**) 15 minutes after the administration of GlcN (2.5 g/kg, IV). (**c**) and (**d**) shows the zoomed-in fitted pools separated from that correspond to (**a**) and (**b**) respectively (R^2^ > 0.99; residual errors < 2%).

Representative GlcN CEST MRI parametric images of another two mice brains are shown in Fig. 2. T_2_ anatomical images (Fig. 2a,d) and overlay MTRasym maps at 1.5ppm (from the water signal) before (Fig. 2b,e) and after GlcN treatment (Fig. 2c,f) depicted a clear increase in the CEST contrast in the brain. The box plots in Fig. 2g show a comparison of the MTRasym (hydroxyls) in the cortex, hippocampus, thalamus, and striatum over the 6 examined mice, before and after GlcN treatment. Following GlcN injection, the net ∆MTRasym signal (delta between GlcN treatment and baseline) at 1.5 ppm (N=6) was − 3.0±1.21%, 3.1±1.65%, 3.0±1.67%, 2.8±1.81% in the cortex, hippocampus, thalamus, and striatum, respectively. Based on One-way ANOVA statistical analysis, there was a significant difference in MTRasym at the baseline and almost 40 min after GlcN injection in the cortex (P=0.0128), hippocampus (P=0.0468), thalamus (P=0.0142). According to the Tukey HSD test, the means MTRasym of each of those examined brain area were significantly different before vs. after treatment. No major differences were found in the striatum (P=0.0764). Figure 3 depicts the average spectral data of the brain cortex as shown using Z-spectra for the six mice examined (using identical ROIs as in the MTRasym analysis). To account for the contribution of each metabolite, a multi-pool Lorentzian fitting approach was applied. Figure 3 shows an increase in the hydroxyl (OH) and amine/amide CEST signals, as well as in the rNOE signal, in the cortex after 15 min. of GlcN treatment, demonstrating GlcN uptake in the brain. The integrals for the hydroxyl, amine/amide CEST, and rNOE pools in the cortex were increased by 18%, 39% and 30% respectively, with 95% confidence bounds.

**Figure 4:**
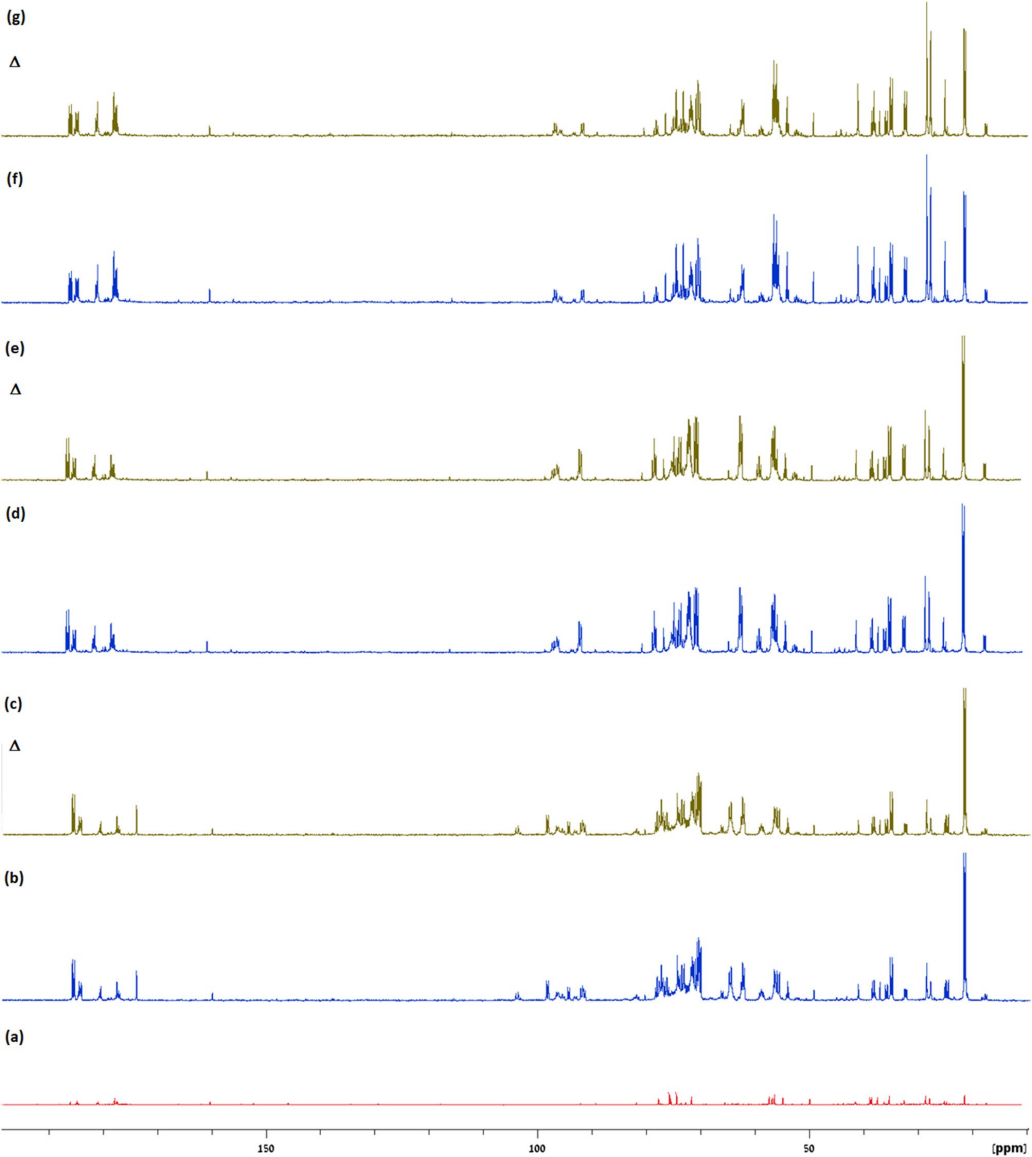
^1^H‐decoupled ^13^C NMR spectra of pooled extracts from brains of mice (**a**) untreated (n=5), (**b**) 15 min after treatment with [UL‐^13^C] ‐GlcN·HCl (2 g/kg IV, n=5), (**d**) 30 minutes after treatment with [UL‐^13^C] ‐GlcN·HCl (2 g/kg IV, n=5), (**f**) 60 min after treatment with [UL‐^13^C] ‐GlcN·HCl (2 g/kg IV, n=5), and the (**c**,**e**,**g**) corresponding net* signal of GlcN and its metabolites in the brains, respectively (B_0_=11.7T, T=25°C, pH=7.4). *The green lines represent the difference between the treatment and the baseline.

**Figure 5:**
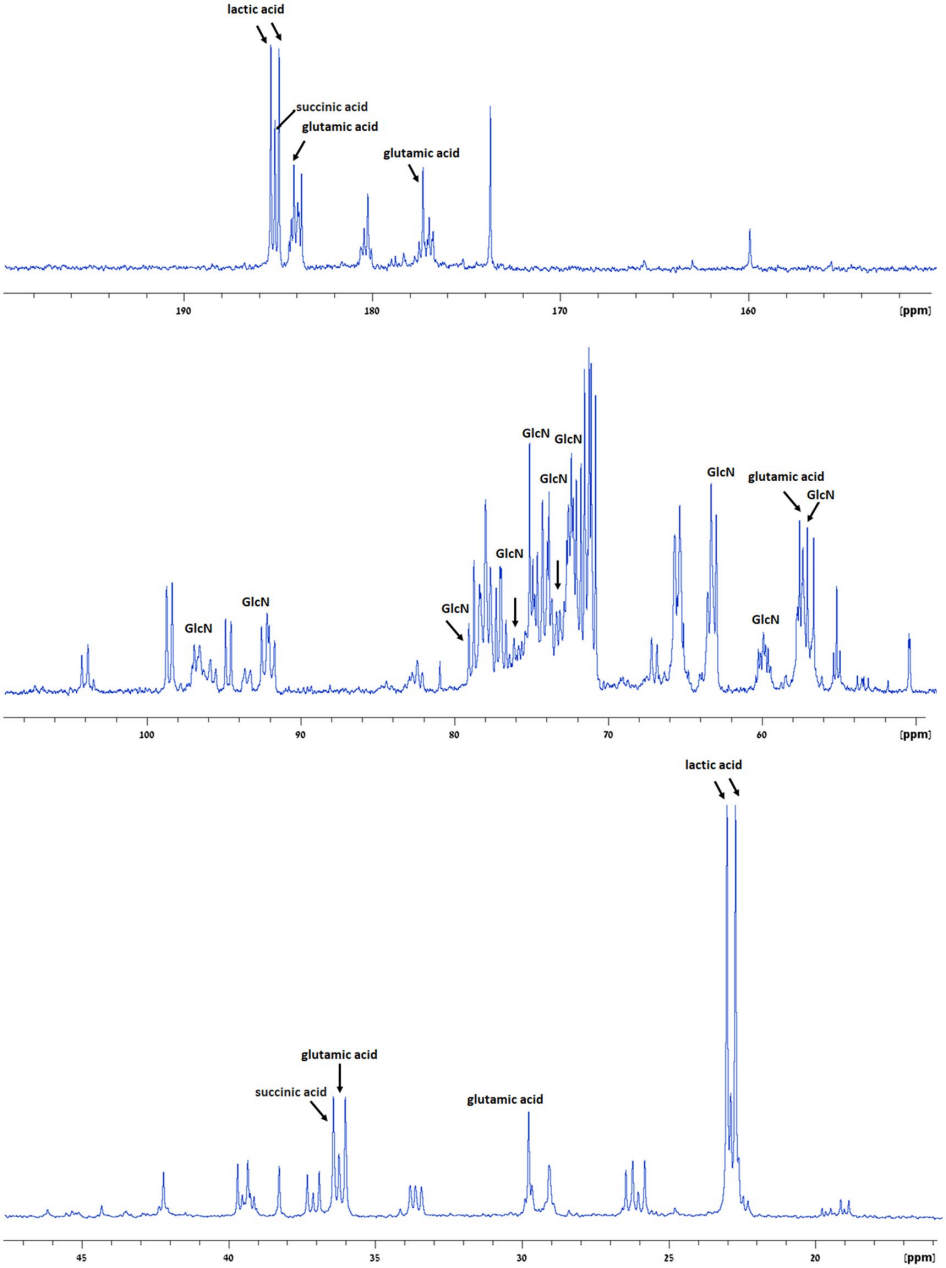
^1^H-decoupled ^13^C NMR spectra of pooled brains extract 15 minutes after [UL-^13^C] -GlcN·HCl treatment (2 g/kg IV, n = 5) with the assignments of GlcN and main organic acids peaks.

To further confirm the specific contributions of metabolites to the CEST contrast (Figs. 1, 2, 3), high-resolution ^13^C NMR spectroscopy studies were performed on mice brain extracts, before and after IV administration of [UL-^13^C]-GlcN.HCl (2.5 g/kg). Figure 4 displays representative ^13^C NMR results of pooled extracts of mice brains taken from untreated mice (Fig. 4a), treated mice 15, 30, 60 minutes post GlcN administration (Fig. 4b,d,f, respectively). Each group was composed of 5 mice. The net increased signals of GlcN and its metabolites in the brain following GlcN administration is shown in Fig. 4c,e,g. Previously reported data provided full spectra assignment^23^. The signals of GlcN and lactic, glutamic, and succinic acids are marked in Fig. 5. The GlcN signals are increased at 15 and 30 min but decreased at 60 minutes. At 15 minutes after GlcN treatment (Figs. 4b,c and 5) there was a marked increase in lactic acid, which appears as doublets at 22.6, 22.96 and 184.9, 185.4 ppm as a result of ^13^C-^13^C J coupling. This signal was slightly decreased at 60 min. The other peak around 71.3 ppm is not well resolved from other peaks. Other organic acids such as succinic (at 36.9 185.2 ppm) and glutamic (at 29.7, 36.3, 57.5, 177.3 and 184.1 ppm) acids were also observed. A large number of peaks were assigned to N-Acetyl-D-glucosamine (GlcNAc), N-acetyl-D-glucosamine-1-phosphate (GlcNAc-1P), N-acetyl-D-glucosamine-6-phosphate (GlcNAc-6P) and uridine diphosphate-N-acetylglucosamine (UDP-GlcNAc). The last compound is a precursor of all macromolecules containing amino sugars^26^, which is involved during more advanced steps of GlcN metabolic pathways.

The peaks assignment was according to previously published data for each metabolite separately^23^ and based on Madison Metabolomics Consortium Database^49^ and Biological Magnetic Resonance Data Bank^50^.

## Discussion

To the best of our knowledge, this is the first report of observation by CEST MRI of uptake of GlcN and its metabolism in the brain. A significant CEST MRI signal increase over the entire mouse brain was observed following the IV administration of GlcN. The most pronounced alterations were detected in the cortex, hippocampus, and thalamus.

The findings imply that CEST signals derived from GlcN *metabolites* are the primary source of this effect. This conclusion is supported by two separate methodologies: (i) Multi-pool Lorentzian fitting of Z-spectra- which revealed that the majority of the increased CEST signal originates from the amine/amide and rNOE pools (instead of the hydroxyl dominant signal reflected in the Z spectrum of free GlcN^18^). (ii) ^13^C NMR spectroscopy of brains extracts treated with [UL-^13^C]-GlcN (Fig. 4)- which revealed a variety of metabolites in addition to GlcN contribution seen in extracts taken after [UL-^13^C]-GlcN administration (Fig. 5). One should note that the brain extracts contain soluble metabolites but not proteins. Thus, the accumulation of macromolecular metabolic products containing GlcN cannot be detected in the brain extracts but is revealed by in vivo observation of rNOE. One of the identified metabolites, UDP-GlcNAc, serves as a precursor for macromolecules containing amino sugars such as proteoglycans, glycoproteins, and glycolipids^26^. Additionally, GlcN can account for up to 25% of covalently linked sugar monomers in brain glycogen, a notably higher proportion compared to muscle and liver glycogen (1% and 0.1%, respectively)^49^. According to Sun RC et al.^51^, GlcN is utilized in the brain in three different ways: as a free metabolite, glycogen-bound, or to produce GlcNAc in branched glycans and poly-mannose chains; the interaction between these three GlcN compartments is critical to maintaining homeostatic brain metabolism. As GlcN is involved in protein formation, and can be metabolically integrated into cell surface glycoproteins, a possible GlcN interaction with proteins may result in binding^52,53^, leading to the expansion of the CEST profile and resulting in more separated chemical shifts from the water resonance. Thus, the profile of the MTRasym shown in Fig. 1 is centered around 1.5−2.0 ppm, which may allow for a better CEST clinical examination setting. Note that MTRasym plots (Fig. 1) exhibit low or zero intensity beyond 3 ppm, where the Z spectra (Fig. 3) indicates relatively high intensity. This is the result of compensation from the rNOE, centered around -3 ppm (Fig. 3). Again, the broad averaged Z spectra shown in Fig. 3 can be explained by the ^13^C NMR analysis of the brain extracts (Figs. 4, 5), showing a variety of metabolites that can contain hydroxyl, amine and amide groups that can contribute to the total CEST MRI signal.

Among the metabolites observed in the ^13^C NMR spectra of brain extracts following administration of [UL‐^13^C] ‐GlcN·HCl (Figs. 4, 5) there is a conspicuous contribution of organic acids. While lactic acid is most prominent, other acids include glutamic and succinic acids. An increase in organic acids following GlcN treatment has also been reported in tumors extracts^23^. The presence of these acids may result in reduced pH. This may increase the measured CEST signal, as reduced pH will result in reduced hydroxyl proton exchange rate, improving its detection^18^.

It should be noted that the high sensitivity of CEST imaging renders it useful even in subtle alterations caused by GlcN uptake; however, CEST specificity should be further investigated, as the pathology-relevant background signal may mask and overlay the net effect observed for a specific application, which requires the accumulation of GlcN with sufficient concentration. In this context, although GlcN can be administrated at relatively high doses (due to its excellent safety profile), the minimum GlcN detectable dose should be established for a specific biomedical task, to avoid background signal interference.

## Conclusions

This work demonstrates that GlcN brain uptake can be detected using CEST MRI and that the CEST signal is mainly due to GlcN metablites. It is customary to categorize the various CEST MRI applications to endogenous and exogenous CEST MRI^54^. Based on the present results we may suggest a new category, namely Metabolic Products-CEST MRI. In the future, the method should be further explored in brain disease models to better understand and test its potential. As it can cross the BBB, GlcN has the potential to serve as a safe MRI contrast agent for brain diseases detection and monitoring.

## Data Availability

The datasets generated and analyzed during the current study are available from the corresponding author on reasonable request.
